# Using DNA barcoding to differentiate invasive *Dreissena* species (Mollusca, Bivalvia)

**DOI:** 10.3897/zookeys.365.5905

**Published:** 2013-12-30

**Authors:** Jonathan Marescaux, Karine Van Doninck

**Affiliations:** 1 Laboratory of Evolutionary Genetics and Ecology, Research Unit in Environmental and Evolutionary Biology, Department of Biology, University of Namur, 61 rue de Bruxelles, 5000 Namur, Belgium

**Keywords:** COI, zebra mussel, quagga mussel, barcoding gap, RFLP

## Abstract

The zebra mussel (*Dreissena polymorpha*) and the quagga mussel (*Dreissena rostriformis bugensis*) are considered as the most competitive invaders in freshwaters of Europe and North America. Although shell characteristics exist to differentiate both species, phenotypic plasticity in the genus *Dreissena* does not always allow a clear identification. Therefore, the need to find an accurate identification method is essential. DNA barcoding has been proven to be an adequate procedure to discriminate species. The cytochrome *c* oxidase subunit I mitochondrial gene (COI) is considered as the standard barcode for animals. We tested the use of this gene as an efficient DNA barcode and found that it allow rapid and accurate identification of adult *Dreissena* individuals.

## Introduction

Biological invasions are a topical issue in today’s world since they are the biggest threat to biodiversity after habitat destruction. The first, and probably the biggest, problem for scientists is to deal with widely divergent perceptions of the criteria defining “invasive” species (Colautti and MacIsaac 2004). In the management and policy field, such species are defined as “alien species whose introduction does, or is likely to, cause economic or environmental harm or harm to human health” (Invasive Species Advisory Committee 2006). By cons, from a strict scientific point of view, an invasive species is “an exotic species that present a tendency to spread with high densities from its point of introduction” (Vermeij 1996, Beisel and Lévêque 2010). A second problem for both scientists and managers is to rapidly characterize a new invasion.

The zebra mussel (*Dreissena polymorpha* (Pallas, 1771) and the quagga mussel (*Dreissena rostriformis bugensis* Andrusov, 1897) are invasive freshwater bivalves in Europe and North America (Mills et al. 1996, Son 2007). Both species are native to the Ponto-Caspian area (Son 2007) and have major negative ecological and economic impacts such as biofouling and food web alteration (Sousa et al. 2013). Several studies have shown that the newly introduced quagga mussel can often dominate well-established zebra mussel populations within only a few years and even outcompete it in some cases (Wilson et al. 2006, Heiler et al. 2012). Wilke et al. (2010) showed that, in addition to the well-known zebra and quagga mussels, two others *Dreissena* species native to the Balkans (*Dreissena presbensis* (Kobelt, 1915) and *Dreissena blanci* Westerlund, 1890) begin to expand in the area and may be potentially invasive in Europe.

Although *Dreissena* specialists may discriminate adults of the different species based on internal and external shell features (Pathy and Mackie 1993, Mills et al. 1996, Sablon et al. 2010), this task remains difficult for managers. It becomes even more problematic when identifying larvae, which is the most invasive form of *Dreissena* (Marescaux et al. 2012a, b). For example, the invasion of the Meuse River in Belgium by the quagga mussel remained undetected because Belgian national agencies never made the distinction with the zebra mussel. Therefore, tools for rapid identification of both adult specimens and larvae are needed in order to detect newly invaded habitats. DNA barcoding has been proven to be an effective method both for species detection and to assign new specimens to already identified species (Hebert et al. 2003a, Birky et al. 2010). Here we amplified part of the cytochrome *c* oxidase subunit I (COI) mitochondrial gene, the most-widely utilized gene for animal DNA barcoding (Consortium for the Barcode of Life 2013) and we tested four delimitation metrics to differentiate *Dreissena* species. We also demonstrate that restriction fragment length polymorphism (RFLP) could be used as an inexpensive method to distinguish between zebra and quagga mussel.

## Methods

### Samples collection

*Dreissena* samples were collected in the Meuse River (see Marescaux et al. 2012a, b for sampling protocol and locations). The mussels were collected in the littoral zone of the river bank from stones which were picked up manually from a depth of 30–40 cm.

### COI sequencing

Total genomic DNA was extracted from 241 *Dreissena* individuals using the «DNeasy Blood and Tissue» kit (Qiagen) according to manufacturer guidelines. To minimize cost, DNA extraction with the CTAB (hexadecyltrimethylamoniumbromide) protocol proposed by Winnepenninckx et al. (1993) could also be used. A fragment of 654 base pairs (bp) of the COI mitochondrial gene was amplified using universal primers (Folmer et al. 1994). Amplifications were performed in 25 μl total volume including 0.5 or 1 μl of gDNA, 1 × GoTaq Green reaction buffer (Promega), 200 μM of dNTPs (Promega), 0.5 μM of both primers and 0.1 U of GoTaq DNA polymerase (Promega). PCR cycling conditions were as follows: an initial step of 94 °C for 4 min, followed by 30 cycles of 94 °C for 45 s, 45 °C for 45 s and 72 °C for 45 s, and then a final extension of 72 °C for 10 min. DNA sequencing was performed by the Genoscreen Company (France). Sequences were visualized and aligned using BioEdit v7.0.5.3 (Hall 1998).

### Phylogenetic analysis

Sequences were collapsed into unique haplotypes using DnaSP (Librado and Rozas 2009). In order to determine the number of *Dreissena* species in the Meuse River we tested three barcoding methods: (i) the “Operational Taxonomic Units” (OTU) (Hebert et al. 2003a), (ii) the “Automatic Barcode Gap Discovery” (ABGD) (Puillandre et al. 2012), and (iii) the “K/θ method” (4 × rule) (Birky et al. 2010). The K/θ method specifies that if the genetic distance between clusters is higher than 4 times the genetic distance within the cluster then species are distinct (Birky et al. 2010, Tang et al. 2012). Neighbour-Joining (NJ) trees and matrix of pairwise distances were calculated using the Kimura 2-parameter (K2P) model and were generated using MEGA4 in order to define OTU’s (Tamura et al. 2007). Sequences found in GenBank (Table 1) were used to construct a haplotype network using Network v4.6 (Bandelt et al. 1999).

**Table 1.** GenBank accession numbers and localities of *Dreissena* spp. sequences included in the network analysis.

GenBank	Taxon	Location
DQ840122	*Dreissena polymorpha polymorpha*	Black and Caspian Seas
DQ840125	*Dreissena polymorpha polymorpha*	Liman, Caspian Sea
DQ840123	*Dreissena polymorpha polymorpha*	Caspian Sea
DQ840121	*Dreissena polymorpha polymorpha*	Black and Caspian Seas
EF414493	*Dreissena polymorpha*	Turkey
U47653	*Dreissena polymorpha*	Lake Ontario
AF474404	*Dreissena polymorpha*	Poland
EU484441	*Dreissena polymorpha*	Lake Superior
EU484437	*Dreissena polymorpha*	Lake Superior
EU484448	*Dreissena polymorpha*	Lake Superior
EU484444	*Dreissena polymorpha*	Lake Superior
AM748997	*Dreissena polymorpha*	Italy
AM748986	*Dreissena polymorpha*	Germany
AM748977	*Dreissena polymorpha*	Italy
U47651	*Dreissena bugensis*	Lake Ontario
U47650	*Dreissena bugensis* var. *profunda*	Lake Ontario
DQ840132	*Dreissena bugensis*	Black Sea
EF080861	*Dreissena rostriformis bugensis*	Hollandsch Diep
AF495877	*Dreissena bugensis*	Ukraine
AF479637	*Dreissena bugensis*	Ukraine
AM748999	*Dreissena polymorpha*	Germany

### Restriction fragment length polymorphism analysis (RFLP)

Using the *restriction map* application (http://www.bioinformatics.org/sms2/rest_map.html), we selected two endonucleases to differentially cut the COI gene of *Dreissena* species: Hinf I and Nla III. We also tested two other enzymes used in previous studies: Nla IV (Baldwin et al. 1996) and Scr FI (Claxton et al. 1998).

Restriction analysis of the amplified 654 bp COI fragment was carried out on each dreissenid haplotype (using individuals from the Meuse River). For each haplotype, the RFLP was performed in 31 μl total volume including 10 µl of PCR reaction mixture, 18 µl of distilled water, 2 µl of buffer (supplied by the manufacturer with the enzyme), and 1 µl of enzyme. Digests were incubated at 37 °C for 3 hours and then loaded on 2% agarose gels.

## Results

**Figure 1.**
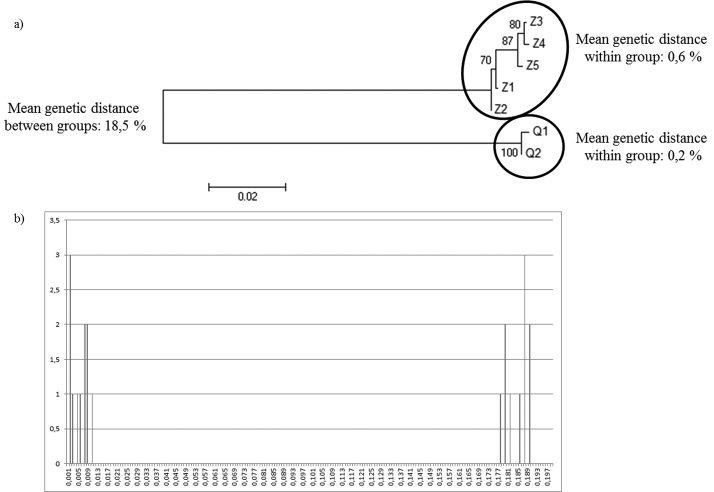
Barcoding analysis based on a fragment of 654 base pairs of the COI gene. **a**) NJ analysis of K2P-pairwise distances **b**) “barcoding gap” method based on the K2P-pairwise distance.

Sequencing of the 654 bp COI fragment revealed seven haplotypes among the 241 *Dreissena* individuals. The OTU method revealed, by a NJ tree, two clusters separated by a genetic distance of 18.5% (Figure 1a), which is higher than the 3% threshold typically used for species delimitation with COI (Hebert et al. 2003b). This first analysis, therefore, suggests the occurrence of two species. We obtained the same results with the ABGD method. Indeed, the K2P-distances show two distinct clusters (Figure 1b). One cluster formed by haplotype 1 and 2, and a second cluster containing the five other haplotypes, all corresponding to those separated in the tree. Moreover, the genetic distances within our two clusters (0.6% and 0.2%, respectively) are four times lower than the genetic distance between them (18.5%) (Figure 1a) confirming the presence of two *Dreissena* species.

**Figure 2.**
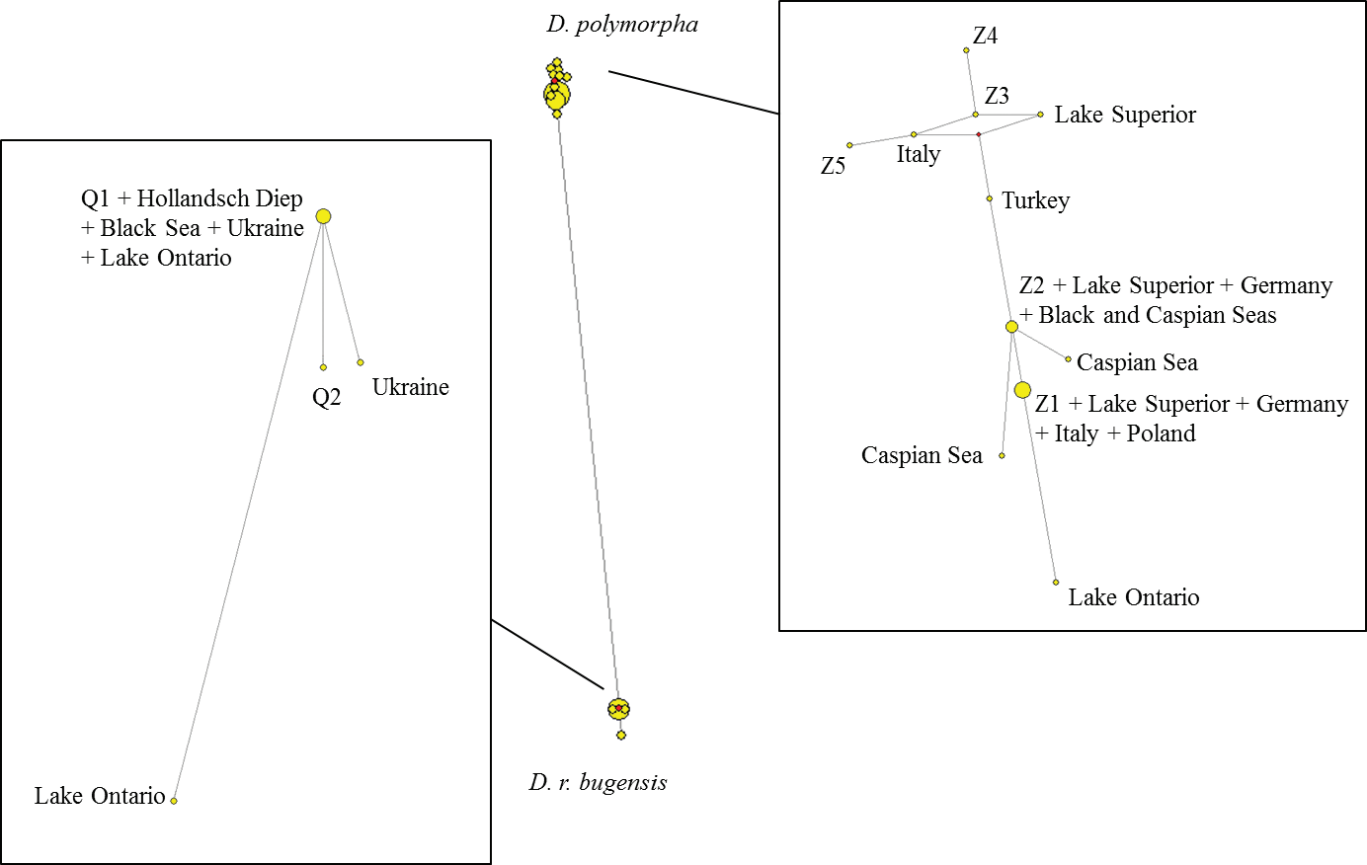
Haplotype networks based on a fragment of 654 base pairs of the COI gene. Our seven haplotypes are labelled: Q1 and Q2 for haplotypes 1 and 2 (belonging to *Dreissena rostriformis bugensis*) / Z1 to Z5 for the 5 other haplotypes (belonging to *Dreissena polymorpha*).

Our network (Figure 2) revealed that haplotypes 1 and 2 (Q1 and Q2) cluster with *Dreissena rostriformis bugensis* and the five other haplotypes (Z1 to Z5) cluster with *Dreissena polymorpha*. This, together with the three barcoding methods which each identified two clusters, shows that both *Dreissena polymorpha* and *Dreissena rostriformis bugensis* species occur in the Meuse River.

**Figure 3.**
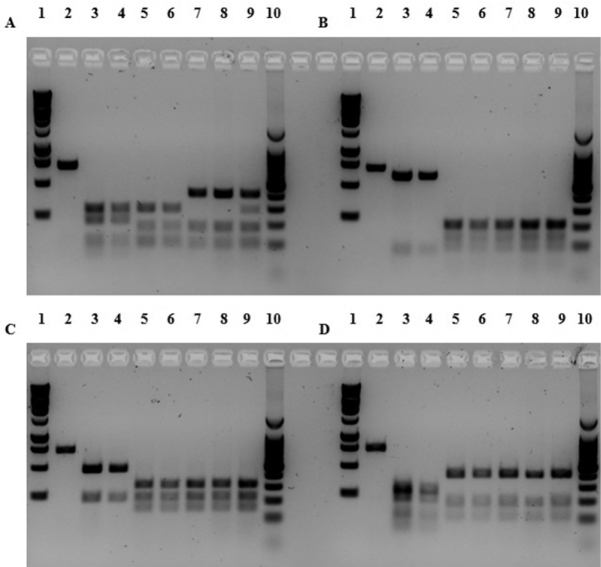
RFLP analysis of the COI gene to distinguish *Dreissena rostriformis bugensis* (Q haplotype) and *Dreissena polymorpha* (Z haplotype) using the endonucleases (**A**) Nla IV (**B**) Hinf I (**C**) Nla III and (**D**) Scr FI. Lane 1, 1-kb ladder; lane 2, non-digested fragment of quagga mussel; lane 3, Q1 haplotype; lane 4, Q2 haplotype; lane 5, Z1 haplotype; lane 6, Z2 haplotype; lane 7, Z3 haplotype; lane 8, Z4 haplotype; lane 9, Z5 haplotype; lane 10, 100-bp ladder.

Digestion profiles for each haplotype are illustrated in Figure 3. Each of the four endonucleases tested, yielded distinct restriction patterns between both *Dreissena* species. Digestion with Nla IV produced four fragments in quagga mussels (Q haplotype) of approximately 70, 79, 211, and 294 bp and three distinct patterns for the zebra mussel (Z haplotype): haplotype Z1 and Z2 (91, 120, 150, and 293 bp), haplotype Z3 and Z4 (91, 150, and 413 bp), and haplotype Z5 (91, 150, 200, and 413 bp). We suggest here that the 200 bp fragment of the haplotype Z5 is an artefact, as confirmed by the restriction map, since the summed fragment lengths do not add up to the expected 654 bp. We infer that haplotype Z5 has the same pattern as haplotype Z3 and Z4. Digestion with Hinf I produced two fragments in quagga mussels of approximately 73 and 581 bp and five fragments in zebra mussels of approximately 31, 101, 114, 195, and 213 bp. The small fragments can not be distinguished on the gel but the difference between quagga and zebra is clear. Digestion with Nla III produced two fragments in quagga mussels of approximately 193 and 461 bp and three fragments in zebra mussels of approximately 193, 319, and 335 bp. Digestion with Scr FI produced five fragments in quagga mussels of approximately 42, 53, 120, 171, and 268 bp and three fragments in zebra mussels of approximately 95, 152, and 407 bp. The digestion pattern for the quagga mussel using the endonuclease Scr FI is not clearly defined (smear) since the five fragments are very short.

## Discussion

On September 9 2013, the European Commission has published a proposal for a Regulation on the prevention and management of the introduction and spread of invasive alien species. This proposal highlights three types of interventions: prevention, early warning and rapid response, and then management of invasive species (European Commission 2013). In this context, rapid identification methods are needed to detect invasive species in periodic surveys, e.g. inspection of ballast water. We showed in previous work (Marescaux et al. 2012a, b) that visual identification and morphometric analyses are not always sufficient to differentiate both zebra and quagga mussel probably due to phenotypic plasticity. This is particularly true for larval identification. In addition, two other *Dreissena* species may become invasive and should be detected promptly.

In order to help managers and national agencies, we propose here the use of the COI mitochondrial gene as a barcode to discriminate *Dreissena polymorpha* and *Dreissena rostriformis bugensis*. Moreover, it is possible to conduct a RFLP analysis on this gene to obtain results without sequencing cost. This method could also easily be applied to *Dreissena presbensis* and *Dreissena blanci* since the COIgene have already been sequenced by Albrecht et al. (2007) and Wilke et al. (2010) and sequences are available on GenBank (accession numbers EF414478–EF414492, EF414496, HM209829–HM210081). We showed that the endonuclease Nla IV, previously used by Baldwin et al. (1996), presents different restriction patterns for the zebra mussel haplotype and not a clear distinction between some zebra mussel haplotypes (Z1 and Z2) and the quagga mussel haplotypes. Therefore, we do not recommend the use of this enzyme to discriminate between quagga and zebra mussel. The three other endonucleases tested during this study present a clear distinction between both species despite the fact that a smear appears using endonucleases Hinf I and Scr FI. Moreover, Nla III and Scr FI will produce a unique RFLP banding pattern for *Dreissena blanci* and *Dreissena presbensis* different from those observed in the zebra and quagga mussel.

This study is the first step of an extensive phylogeographical analysis on the invasion of Western Europe by the dreissenids. Further experiments will be needed to assess potential risks of both zebra and quagga mussels on native biodiversity in Western European rivers, e.g. predation on phytoplankton, infestation on native bivalves and alteration of macro-invertebrate communities.
